# Supplementary material for: The significance of information variables in polydrug use by adolescents: insights from a cross-sectional study in Tarragona (Spain)
Source: PeerJ. 2024 Jan 19;12:e16801. doi: 10.7717/peerj.16801 (PMC10802159; doi:10.7717/peerj.16801)
Supplement: Supplemental Information 2 [file peerj-12-16801-s002.docx]

I live with is answered as…

| I live with my mother and my mother [1] | |
| --- | --- |
| I live with my mother, but not with my father [2] | |
| I live with my father, but not with my mother [3] | |
| I live with my mother and her partner [4] | |
| I live with my father and his partner [5] | |
| I live with my grandparents (or grandfather alone, or grandmother alone) [6] | |
| I live with friends [7] | |
| I live alone [8] | |
| I live in other situations (foster family, children's home, etc.) | |
| [9] N.C. (Not classified) [99] | |
|  | |
| I was born, my father was born and my mother was born is answered as [1] Spain, [2] Abroad. | |
| Q1 is 0 male and 1 female  Q2 is a numeric variable | |
| In the case of Q3, Q5 and Q8 (1) Completely disagree (2) Mostly disagree (3) Neither agree nor disagree (4) Mostly agree and (5) Completely agree. |  |
| In the case of Q4, (1) almost never; (2) rarely; (3) sometimes; (4) often; (5) almost always. |  |
| In the case of Q6: (1) very difficult, (2) difficult, (3) easy; (4) very easy. |  |
| In the case of Q7, (1) Does not apply at all to me; (2) Does not apply well to me; (3) Applies quite well to me; (4) Applies very well to me. |  |

Q9, information level is answered from 1 very bad informed from 5 very well onformed

From Q10 to Q15 the variables are 1 if the source of information is reported and 0 otherwise
