# Supplementary material for: The significance of information variables in polydrug use by adolescents: insights from a cross-sectional study in Tarragona (Spain)
Source: PeerJ. 2024 Jan 19;12:e16801. doi: 10.7717/peerj.16801 (PMC10802159; doi:10.7717/peerj.16801)
Supplement: Supplemental Information 4 [file peerj-12-16801-s004.pdf]

Questions in English

| **Input questions** |
|---|
| Q1: What is your sex? |
| Q2: What is your age? |
| Q3: Non adherence to norms |
| NORMS.1: Most rules can be broken if they are not convenient.<br>NORMS.2: I follow the rules that I want to follow.<br>NORMS.3: It is hard to trust anything because everything changes.<br>NORMS.4: In fact, no one knows what is expected of him/her in life.<br>NORMS.5: You can never be sure of anything in life.<br>NORMS.6: Sometimes, it is necessary to break the rules to succeed.<br>NORMS.7: Following the rules does not guarantee success. |
| Q4: AGRESSIVE |
| AGRESSIVE.1: I have been easily bothered or irritated<br>AGRESSIVE.2: I have had outbursts of anger that I could not control.<br>AGRESSIVE.3: I have wanted to break or damage things.<br>AGRESSIVE.4: I have had a fight with someone.<br>AGRESSIVE.5: I yelled at someone or threw things at them |
| Q5: School support |
| SCHOOLENG.1: The adults at my school care about me.<br>SCHOOLENG.2: I have friends at my educational center who care about me.<br>SCHOOLENG.3: The students at my educational center are kind to each other.<br>SCHOOLENG.4: My educational center is helping me achieve goals that matter to me.<br>SCHOOLENG.5: I enjoy participating in activities at my educational center. |
| Q6: Parental support |
| PARSUPP.1: I receive care and affection from my parents.<br>PARSUPP.2: With my parents, I can talk about personal matters.<br>PARSUPP.3: I receive advice from my parents regarding my studies.<br>PARSUPP.4: I receive advice from my parents regarding other topics (your projects).<br>PARSUPP.5: I also receive help from my parents with other things. |
| Q7: Parental control |
| PARCONT.1: My parents consider it important that my studies go well.<br>PARCONT.2: They establish clear rules about what I can do at home.<br>PARCONT.3: hey establish clear rules about what I can do outside the house.<br>PARCONT.4: They establish clear rules about when I have to be home in the evening.<br>PARCONT.5: They know who I am with at night.<br>PARCONT.6: They know where I am at night.<br>PARCONT.7: They know my friends.<br>PARCONT.8: They know the parents of my friends. |
| Q8: Peer Influence |
| PEERINFL.1: Sometimes you have to smoke cigarettes to avoid being left out of the peer group.<br>PEERINFL.2: Sometimes it is necessary to drink alcohol to avoid being left out of the peer group.<br>PEERINFL.3: Sometimes it is necessary to smoke cannabis to avoid being left out of the peer group.<br>PEERINFL.4: Sometimes it is necessary to skip classes to avoid being left out of the peer group. |
| Q9: INF_LEVEL |
| |
| My information about |

| |
|---|
| substance use come from: |
| Q10: School |
| Q11: Parents/legal guardians |
| Q12: Mass media |
| Q13: Internet |
| Q14: Siblings |
| Q15: Peers and friends |
| Number of sources |
| Monitored sources (Q10+Q11+Q12) |
| No monitored sources (Q13+Q14+Q15) |
| Prevalence last 30 days |
| Use 1: Did you used alcohol drinks last 30 days? |
| Use2: Did you used cigarettes last 30 days? |
| Use 3: Dis you used cannabis last 30 days? |
