# Supplementary material for: The significance of information variables in polydrug use by adolescents: insights from a cross-sectional study in Tarragona (Spain)
Source: PeerJ. 2024 Jan 19;12:e16801. doi: 10.7717/peerj.16801 (PMC10802159; doi:10.7717/peerj.16801)
Supplement: Supplemental Information 5 [file peerj-12-16801-s005.docx]

Preguntas de entrada

Q1: ¿Cuál es tu sexo?

Q2: ¿Cuál es tu edad?

Q3: No adherencia a normas

NORMS.1: La mayoría de las reglas se pueden romper si no son convenientes.

NORMS.2: Sigo las reglas que quiero seguir.

NORMS.3: Es difícil confiar en algo porque todo cambia.

NORMS.4: De hecho, nadie sabe qué se espera de él/ella en la vida.

NORMS.5: Nunca puedes estar seguro de nada en la vida.

NORMS.6: A veces, es necesario romper las reglas para tener éxito.

NORMS.7: Seguir las reglas no garantiza el éxito.

Q4: AGRESIVO

AGRESSIVE.1: Me he sentido fácilmente molesto o irritado.

AGRESSIVE.2: He tenido arrebatos de ira que no podía controlar.

AGRESSIVE.3: He querido romper o dañar cosas.

AGRESSIVE.4: He tenido una pelea con alguien.

AGRESSIVE.5: Le grité a alguien o le lancé cosas.

Q5: Apoyo escolar

SCHOOLENG.1: Los adultos en mi escuela se preocupan por mí.

SCHOOLENG.2: Tengo amigos en mi centro educativo que se preocupan por mí.

SCHOOLENG.3: Los estudiantes en mi centro educativo son amables entre ellos.

SCHOOLENG.4: Mi centro educativo me está ayudando a alcanzar metas que son importantes para mí.

SCHOOLENG.5: Disfruto participando en actividades en mi centro educativo.

Q6: Apoyo parental

PARSUPP.1: Recibo cuidado y afecto de mis padres.

PARSUPP.2: Con mis padres, puedo hablar sobre asuntos personales.

PARSUPP.3: Recibo consejos de mis padres sobre mis estudios.

PARSUPP.4: Recibo consejos de mis padres sobre otros temas (tus proyectos).

PARSUPP.5: También recibo ayuda de mis padres con otras cosas.

Q7: Control parental

PARCONT.1: Mis padres consideran importante que me vaya bien en mis estudios.

PARCONT.2: Establecen reglas claras sobre lo que puedo hacer en casa.

PARCONT.3: Establecen reglas claras sobre lo que puedo hacer fuera de casa.

PARCONT.4: Establecen reglas claras sobre cuándo debo estar en casa por la noche.

PARCONT.5: Saben con quién estoy por la noche.

PARCONT.6: Saben dónde estoy por la noche.

PARCONT.7: Conocen a mis amigos.

PARCONT.8: Conocen a los padres de mis amigos.

Q8: Influencia de amigos

PEERINFL.1: A veces es necesario fumar cigarrillos para no quedar excluido del grupo de amigos.

PEERINFL.2: A veces es necesario beber alcohol para no quedar excluido del grupo de amigos.

PEERINFL.3: A veces es necesario fumar cannabis para no quedar excluido del grupo de amigos.

PEERINFL.4: A veces es necesario faltar a clases para no quedar excluido del grupo de amigos.

Q9: NIVEL_INFO

Mi información sobre el uso de sustancias proviene de:

Q10: Escuela

Q11: Padres/tutores legales

Q12: Medios de comunicación

Q13: Internet

Q14: Hermanos

Q15: Amigos y compañeros

Número de fuentes monitoreadas (Q10+Q11+Q12)

Número de fuentes no monitoreadas (Q13+Q14+Q15)

Prevalencia en los últimos 30 días

Uso 1: ¿Consumiste bebidas alcohólicas en los últimos 30 días?

Uso 2: ¿Consumiste cigarrillos en los últimos 30 días?

Uso 3: ¿Consumiste cannabis en los últimos 30 días?


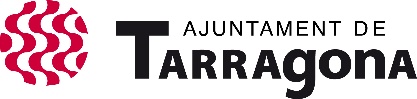

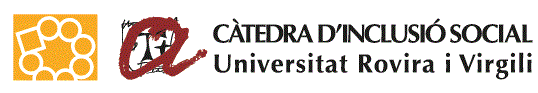


**Encuesta Condiciones de Vida**

**de los y de las Adolescentes de Tarragona**

**2023**

**A los y las estudiantes**

La siguiente encuesta contiene preguntas que se refieren a tus opiniones sobre diversos temas y diferentes tipos de actividades que puedes o no realizar. Tus respuestas son muy importantes. Este cuestionario es completamente diferente a los exámenes, puesto que ninguna respuesta es más correcta que otras. Lo único importante aquí es que contestes eligiendo la respuesta que mejor se ajuste a tus opiniones o actividades.

El cuestionario es absolutamente anónimo. Nadie que conozcas, ni tus profesores/as, ni padres ni madres, conocidos/as o amigos/as, podrán acceder a tus respuestas ni conocer quién ha respondido a este cuestionario.

La participación en la encuesta es completamente voluntaria. Tus respuestas no te afectarán de ninguna forma negativa. Si no te sientes cómodo/a respondiendo a una pregunta o no quieres responderla, puedes dejarla en blanco.

Gracias por tu colaboración

Angel Belzunegui Eraso

Director de la Encuesta

# **¿A qué centro asistes?** (Elige solo una opción)

INS Compte de Rius 1

INS Pons d’Icart 2

INS Camp Clar 3

INS Tarragona 4

INS Pont del Diable 6

INS Vidal i Barraquer 7

INS Pere Martell 8

INS Cal·lípolis 9

INS Torreforta 10

INS Sant Pere i Sant Pau 11

INS Collblanc 12

INS Martí i Franquès 13

COL Sant Domènec de Guzman 14

COL Lestonnac 15

COL Sant Pau Apòstol 16

COL Roig 17

COL La Salle Torreforta 18

COL Joan XXIII 19

COL Santa Teresa de Jesús 20

COL Sagrat Cor 21

COL Mare de Déu del Carme 22

COL El Carme 23

COL La Salle Tarragona 24

COL Mare Nostrum 25

COL Espallargas 26

NC 99

1. **¿En qué zona de Tarragona vives?** (Elige solo una opción)

Part Alta 1

Tarragona centre 2

Riuclar-Icomar 3

La Floresta 4

La Canonja 5

Bonavista 6

Camp Clar 7

Torreforta 8

Sant Salvador 10

Sant Pere i Sant Pau 11

Barris Marítims 12

Llevant-Cala Romana-Boscos-La Mora 14

NC 99

1. **¿Eres un chico o una chica?** (Elige solo una opción) **SEXO**

Chico 1

Chica 2

NC 9

1. **¿En qué año naciste?** (Elige solo una opción) _____________

**A partir de esta variable se construye la variable EDAD**

1. **¿Cuál es tu grado/clase en el instituto?** (Elige solo una opción) **GRADO**

4º ESO 1

1º de Bachillerato 2

2º de Bachillerato 3

1º ciclos formativos 4

2º ciclos formativos 5

NC 9

1. **Yo vivo con…** (elige la respuesta que más se ajuste a tu situación) **CONVIVENCIA**

Vivo con mi madre y mi madre 1

Vivo con mi madre, pero no con mi padre 2

Vivo con mi padre, pero no con mi madre 3

Vivo con mi madre y su pareja 4

Vivo con mi padre y su pareja 5

Vivo con mis abuelos (o abuelo solo, o abuela sola) 6

Vivo con amigos/as 7

Yo vivo solo/a 8

Vivo en otras situaciones (familia de acogida,

casa de niños, etc).......................................................................9

N.C. 99

1. **¿Cuál es el nivel más alto de estudios cursados de tu madre?** (Elige solo una opción, la que mejor se adapte) **ESTUDIOSMADRE**

No tiene teminado los estudios primarios 1

Estudios primarios obligatorios 2

Estudios secundarios obligatorios 3

Estudios secundarios no obligatorios (bachillerato) 4

Estudios de formación profesional 1º grado 5

Estudios de formación profesional 2º grado 6

Comenzó la universidad pero no acabó 7

Tiene un titulo universitario 8

N.S. 98

N.C. 99

1. **¿Cuál es el nivel más alto de estudios cursados de tu padre?** (Elige solo una opción, la que mejor se adapte)

**ESTUDIOSPADRE**

No tiene teminado los estudios primarios 1

Estudios primarios obligatorios 2

Estudios secundarios obligatorios 3

Estudios secundarios no obligatorios (bachillerato) 4

Estudios de formación profesional 1º grado 5

Estudios de formación profesional 2º grado 6

Comenzó la universidad pero no acabó 7

Tiene un titulo universitario 8

N.S. 98

N.C 99

1. **¿Cuál es la actividad principal de tu madre?** (Elige solo una opción, la que mejor se adapte) **ACTIVIDADMADRE**

Trabaja en casa (se ocupa del hogar) 1

Trabaja a tiempo parcial 2

Trabaja a tiempo completo 3

Trabaja en el estranjero 4

Está en paro 5

Tiene una discapacidad, no puede trabajar 6

Está estudiando 7

Trabaja y estudia 8

Es pensionista o jubilada 9

Ha muerto 10

N.S. 98

N.C. 99

1. **¿Cuál es la actividad principal de tu padre?** (Elige solo una opción, la que mejor se adapte) **ACTIVIDADPADRE**

Trabaja en casa (se ocupa del hogar) 1

Trabaja a tiempo parcial 2

Trabaja a tiempo completo 3

Trabaja en el estranjero 4

Está en paro 5

Tiene una discapacidad, no puede trabajar 6

Está estudiando 7

Trabaja y estudia 8

Es pensionista o jubilada 9

Ha muerto 10

N.S. 98

N.C. 99

1. **¿En qué país has nacido? _________ PAISNACIMIENTO**
2. **¿En qué país ha nacido tu padre? ___________ PAISPADRE**
3. **Y tu madre, ¿en qué país ha nacido? ___________ PAISMADRE**

**RE**

1. **¿A qué religión o comunidad perteneces?** (Elige solo una opción, la que mejor se adapte) **RELIGION**

Católica 1

Evangélica 2

Islam 3

Ortodoxa 4

Otra 5

No me identifico con ninguna religión o iglesia 6

NC 9

1. **¿Tu centro de estudios está en el barrio donde vives?** (Elige solo una opción, la que mejor se adapte) **CENTRO**

Sí 1

No 2

NC 9

1. **¿Cuántos días has estado ausente del centro educativo por enfermedad durante los últimos 30 días?** (Elige una opción en cada categoría) **AUSENCIAENFERMEDAD**

Ningún día 0

1 día 1

2 días 2

3 días 3

4 días 4

5 días 5

6 días o más 6

NC 9

1. **¿Cuántos días has estado ausente del centro educativo porque me he saltado las clases (hacer pellas) durante los últimos 30 días?** (Elige una opción en cada categoría) **ABSENTISMO**

Ningún día 0

1 día 1

2 días 2

3 días 3

4 días 4

5 días 5

6 días o más 6

NC 9

1. **¿Cómo se ajustan a ti las siguientes afirmaciones?** (Elige una opción en cada categoría) **MALESTARESTUDIOS**

|  | ***Se aplica casi siempre a mí*** | ***A menudo se aplica a mí*** | ***A veces se aplica a mí*** | ***Se aplica pocas veces a mí*** | Casi nunca se aplica a mí | No contesta |
| --- | --- | --- | --- | --- | --- | --- |
| Encuentro los estudios sin sentido | 1 | 2 | 3 | 4 | 5 | 0 |
| Estoy aburrido/a con los estudios | 1 | 2 | 3 | 4 | 5 | 0 |
| Estoy poco preparado/a para las clases | 1 | 2 | 3 | 4 | 5 | 0 |
| Creo que no me esfuerzo suficiente en los estudios | 1 | 2 | 3 | 4 | 5 | 0 |
| Me parece que los estudios son demasiado fáciles | 1 | 2 | 3 | 4 | 5 | 0 |
| Me parece que los estudios son demasiado difíciles | 1 | 2 | 3 | 4 | 5 | 0 |
| Me siento mal en el instituto | 1 | 2 | 3 | 4 | 5 | 0 |
| Quiero dejar el instituto | 1 | 2 | 3 | 4 | 5 | 0 |
| Quiero cambiar de instituto | 1 | 2 | 3 | 4 | 5 | 0 |
| Me encuentro mal con los profesores | 1 | 2 | 3 | 4 | 5 | 0 |

A partir de estos items se calcula **SUMAMALESTARESTUDIOS**, que es la suma de las puntuaciones de los items anteriores. A menor puntuación, mayor malestar en los estudiós.

1. **¿En qué medida estás de acuerdo o en desacuerdo con las siguientes afirmaciones?** (Elige una opción en cada categoría) **APOYOCENTRO**

|  | ***Completamente de acuerdo*** | Bastante de acuerdo | Ni de acuerdo ni en desacuerdo | Bastante en desacuerdo | Muy en desacuerdo | No contesta |
| --- | --- | --- | --- | --- | --- | --- |
| Los adultos de mi centro se preocupan por mí | 1 | 2 | 3 | 4 | 5 | 9 |
| Tengo amigos/as en mi centro educativo que se preocupan por mí | 1 | 2 | 3 | 4 | 5 | 9 |
| Los estudiantes de mi centro educativo son amables entre ellos | 1 | 2 | 3 | 4 | 5 | 9 |
| Mi centro educativo me está ayudando a conseguir metas que me importan | 1 | 2 | 3 | 4 | 5 | 9 |
| Disfruto participando en las actividades de mi centro educativo | 1 | 2 | 3 | 4 | 5 | 9 |

A partir de estos items se calcula **SUMAAPOYOCENTRO**, que es la suma de las puntuaciones de los items anteriores. A mayor puntuación, mayor apoyo.

1. **¿Cómo de fácil o difícil es que recibas lo siguiente de tus padres/madres o tutors/es legales?** (Elige una opción en cada categoría) **APOYOPADRES**

|  | *Muy difícil* | *Más bien difícil* | *Más bien fácil* | *Muy fácil* | *No contesta* |
| --- | --- | --- | --- | --- | --- |
| Cuidado y aprecio | 1 | 2 | 3 | 4 | 9 |
| Hablar sobre asuntos personales | 1 | 2 | 3 | 4 | 9 |
| Consejo sobre los estudios | 1 | 2 | 3 | 4 | 9 |
| Consejo sobre otros temas (proyectos) tuyos | 1 | 2 | 3 | 4 | 9 |
| Ayuda con otras cosas | 1 | 2 | 3 | 4 | 9 |

A partir de estos items se calcula **SUMAAPOYOPADRES**, que es la suma de las puntuaciones de los items anteriores. A mayor puntuación, mayor apoyo.

1. **¿Con qué frecuencia vives las siguientes situaciones?** (Elige una opción en cada categoría) **ACOMPAÑAPADRES**

|  | ***Casi nunca*** | Rara vez | A veces | A menudo | Casi siempre | No contesta |
| --- | --- | --- | --- | --- | --- | --- |
| Paso tiempo con mis padres/tutores/as legales fuera del horario escolar los días laborables | 1 | 2 | 3 | 4 | 5 | 9 |
| Paso tiempo con mis padres/tutores/as legales durante los fines de semana | 1 | 2 | 3 | 4 | 5 | 9 |
| Mis padres/tutores/as legales saben donde estoy los sábados por la noche* | 1 | 2 | 3 | 4 | 5 | 9 |

*** Este ítem lo excluimos del anàlisis. Es parecido al item de la pregunta 22.**

1. **¿Cómo de bien se ajustan a ti las siguientes afirmaciones?** (Elige una opción en cada categoría) **CONTROLPARENTAL**

| **Mis padres...** | ***Se aplica muy bien a mí*** | ***Se aplica bastante bien a mí*** | ***Se aplica mal a mi*** | ***Se aplica muy mal a mí*** | No contesta |
| --- | --- | --- | --- | --- | --- |
| Consideran que es importante que me vayan bien mis estudiós | 4 | 3 | 2 | 1 | 0 |
| Establecen normas claras sobre lo que puedo hacer en casa | 4 | 3 | 2 | 1 | 0 |
| Establecen normas claras sobre lo que puedo hacer fuera de casa | 4 | 3 | 2 | 1 | 0 |
| Establecen normas claras sobre cuando tengo que estar en casa por la tarde | 4 | 3 | 2 | 1 | 0 |
| Conocen con quien estoy por la noche | 4 | 3 | 2 | 1 | 0 |
| Saben donde estoy por la noche | 4 | 3 | 2 | 1 | 0 |
| Conocen mis amigos/as | 4 | 3 | 2 | 1 | 0 |
| Conocen a los padres de mis amigos/as | 4 | 3 | 2 | 1 | 0 |

A partir de estos items se calcula **SUMACONTROLPARENTAL**, que es la suma de las puntuaciones de los items anteriores. A mayor puntuación, mayor control.

1. **Indica en qué medida se aplica lo siguiente a tu situación** (Elige una opción en cada categoría) **NIVELSOCIOECO**

|  | ***Casi nunca*** | Rara vez | A veces | A menudo | Casi siempre | No contesta |
| --- | --- | --- | --- | --- | --- | --- |
| Mi familia tiene pocos ingresos económicos | 1 | 2 | 3 | 4 | 5 | 9 |
| Mi familia no puede permitirse tener un coche | 1 | 2 | 3 | 4 | 5 | 9 |
| Mi familia a duras penas tiene dinero suficiente para pagar las necesidades (por ejemplo, comida, vivienda, teléfono) | 1 | 2 | 3 | 4 | 5 | 9 |
| Mi familia no tiene dinero suficiente para pagar las actividades extraescolares que más me gustaria hacer (por ejemplo, practicar intrumentos musicales o deportes) | 1 | 2 | 3 | 4 | 5 | 9 |

A partir de estos items se calcula **SUMAnivelSOCIOECO**, que es la suma de las puntuaciones de los items anteriores. A mayor puntuación, mayor situación de vulnerabilidad social.

1. **¿Cómo se ajustan a ti las siguientes afirmaciones? (Elige una opción) INFLUENCIAIGUALS**

|  | ***Completamente de acuerdo*** | Bastante de acuerdo | Ni de acuerdo ni en desacuerdo | Bastante en desacuerdo | Muy en desacuerdo | No contesta |
| --- | --- | --- | --- | --- | --- | --- |
| A veces hay que fumar cigarrillos para no quedar fuera del grupo de iguales | 5 | 4 | 3 | 2 | 1 | 0 |
| A veces es necesario beber alcohol para no quedar fuera del grupo de iguales | 5 | 4 | 3 | 2 | 1 | 0 |
| A veces es necesario fumar cannabis para no quedar fuera del grupo de iguales | 5 | 4 | 3 | 2 | 1 | 0 |
| A veces es necesario "saltarse" las clases para no quedar fuera del grupo de iguales | 5 | 4 | 3 | 2 | 1 | 0 |

A partir de estos items se calcula **SUMAINFLUENCIAIGUALES**, que es la suma de las puntuaciones de los items anteriores. A mayor puntuación, mayor influencia de los iguales.

1. **¿Cómo de bien describe vuestro estado de ánimo las siguientes situaciones en la última semana?** (Elige una opción) **IRRITABILIDAD**

|  | ***Casi nunca*** | Rara vez | A veces | A menudo | Casi siempre | No contesta |
| --- | --- | --- | --- | --- | --- | --- |
| Me he molestado o irritado fácilmente | 1 | 2 | 3 | 4 | 5 | 9 |
| He tenido estallidos de enfado/rabia que no podía controlar | 1 | 2 | 3 | 4 | 5 | 9 |
| He querido romper o dañar cosas | 1 | 2 | 3 | 4 | 5 | 9 |
| He tenido una pelea con alguien | 1 | 2 | 3 | 4 | 5 | 9 |
| Le grité a alguien o le lancé cosas | 1 | 2 | 3 | 4 | 5 | 9 |

A partir de estos items se calcula **SUMAIRRITABILIDAD**, que es la suma de las puntuaciones de los items anteriores. A mayor puntuación, mayor irritabilidad.

1. **¿Cómo de acuerdo estás con las siguientes situaciones?** (Elige una opción) **ACEPTACIONFISICO**

|  | ***Completamente de acuerdo*** | Bastante de acuerdo | Ni de acuerdo ni en desacuerdo | Bastante en desacuerdo | Muy en desacuerdo | No contesta |
| --- | --- | --- | --- | --- | --- | --- |
| Cuando pienso en el aspecto que tendré en el futuro, estoy contento/a | 5 | 4 | 3 | 2 | 1 | 0 |
| A menudo pienso que soy feo/a y poco atractivo/a | 1 | 2 | 3 | 4 | 5 | 0 |
| Estoy contento/a con mi cuerpo | 5 | 4 | 3 | 2 | 1 | 0 |
| Estoy contento/a con los cambios físicos que se han producido en mi cuerpo durante los últimos años | 5 | 4 | 3 | 2 | 1 | 0 |
| Me siento físicamente fuerte y sano/a | 5 | 4 | 3 | 2 | 1 | 0 |
| Estoy contento/a con mi vida | 5 | 4 | 3 | 2 | 1 | 0 |

A partir de estos items se calcula **SUMAaceptacionfisico**, que es la suma de las puntuaciones de los items anteriores. A mayor puntuación, mayor aceptación del propio físico.

**ATENCIÓN**: El item 26.2 es inverso y se ha tratado como tal (invirtiendo la escala).

1. **¿Hasta qué punto estás de acuerdo o en desacuerdo con las siguientes afirmaciones?** (Elige una opción en cada categoría) **NONORMAS**

|  | ***Completamente de acuerdo*** | Bastante de acuerdo | Ni de acuerdo ni en desacuerdo | Bastante en desacuerdo | Muy en desacuerdo | No contesta |
| --- | --- | --- | --- | --- | --- | --- |
| Se pueden romper la mayoría de reglas si no convienen | 5 | 4 | 3 | 2 | 1 | 0 |
| Sigo las reglas que quiero seguir | 5 | 4 | 3 | 2 | 1 | 0 |
| Es difícil confiar en nada porque todo cambia | 5 | 4 | 3 | 2 | 1 | 0 |
| De hecho, nadie sabe qué se espera de él/ella en la vida | 5 | 4 | 3 | 2 | 1 | 0 |
| Nunca se puede estar seguro de nada en la vida | 5 | 4 | 3 | 2 | 1 | 0 |
| A veces, es necesario romper las reglas para tenir éxito | 5 | 4 | 3 | 2 | 1 | 0 |
| Seguir las reglas no garantiza el éxito | 5 | 4 | 3 | 2 | 1 | 0 |

A partir de estos items se calcula **SUMAdisconformeNormas**, que es la suma de las puntuaciones de los items anteriores. A mayor puntuación, mayor disconformidad normativa.

1. **¿Con qué frecuencia has sufrido alguna de las siguientes molestias físicas o mentales en la última semana?** (Elige una opción) **MALESTAR.**

|  | ***Casi nunca*** | Rara vez | A veces | A menudo | Casi siempre | No contesta |
| --- | --- | --- | --- | --- | --- | --- |
| Nerviosismo | 1 | 2 | 3 | 4 | 5 | 0 |
| Un miedo repentino sin motivo aparente | 1 | 2 | 3 | 4 | 5 | 0 |
| Me sentí tenso/a | 1 | 2 | 3 | 4 | 5 | 0 |
| No tener mucho interés en hacer las cosas | 1 | 2 | 3 | 4 | 5 | 0 |
| Tener poca hambre | 1 | 2 | 3 | 4 | 5 | 0 |
| Me sentí solitario/a | 1 | 2 | 3 | 4 | 5 | 0 |
| Lloré fácilmente o quería llorar | 1 | 2 | 3 | 4 | 5 | 0 |
| Tuve problemas para domir | 1 | 2 | 3 | 4 | 5 | 0 |
| Me sentí triste | 1 | 2 | 3 | 4 | 5 | 0 |
| No estaba emocionado/a para hacer las cosas | 1 | 2 | 3 | 4 | 5 | 0 |
| Fui lento/a o tuve poca energía | 1 | 2 | 3 | 4 | 5 | 0 |
| El futuro parecía desesperanzador | 1 | 2 | 3 | 4 | 5 | 0 |
| Pensé en suicidarme | 1 | 2 | 3 | 4 | 5 | 0 |

**A partir de aquí se genera la variable SUMAMALESTAR.** A mayor puntuación, mayor malestar emocional.

1. **¿Has experimentado alguna de las siguientes situaciones?** (Elige una opción en cada categoría)

|  | ***Sí*** | No | No contesta |
| --- | --- | --- | --- |
| Has sufrido abuso sexual (tú como víctima) ABUSOSEXUAL | 1 | 2 | 9 |
| Has sufrido abuso sexual por parte de un adulto procedente de tu familia ABUSOSEXUAL1 | 1 | 2 | 9 |
| Has sufrido abuso sexual por parte de un adulto que no sea de tu família ABUSOSEXUAL2 | 1 | 2 | 9 |
| Te has sentido discriminado/a por tu expresión y/o identidad de genero DISCRIMINACIONGENERO | 1 | 2 | 9 |

1. **¿Cómo se ajustan a ti las siguientes afirmaciones?** (Elige una opción en cada categoría) **AUTOESTIMA**

|  | ***Completamente de acuerdo*** | Bastante de acuerdo | Ni de acuerdo ni en desacuerdo | Bastante en desacuerdo | Muy en desacuerdo | No contesta |
| --- | --- | --- | --- | --- | --- | --- |
| a) Creo que soy igual de valioso/a como los demás | 5 | 4 | 3 | 2 | 1 | 0 |
| b) Creo que tengo muchas cualidades | 5 | 4 | 3 | 2 | 1 | 0 |
| f) En general me inclino a pensar que soy un fracaso | 1 | 2 | 3 | 4 | 5 | 0 |
| c) Soy capaz de hacer las cosas tan bien como la mayoría de las personas | 5 | 4 | 3 | 2 | 1 | 0 |
| g) Creo que no tengo demasiadas cosas de las que sentirme orgulloso/a | 1 | 2 | 3 | 4 | 5 | 0 |
| d) Tengo una actitud positiva hacia mí mismo/a | 5 | 4 | 3 | 2 | 1 | 0 |
| e) En general, estoy satisfecho/a conmigo mismo/a | 5 | 4 | 3 | 2 | 1 | 0 |
| h) Me gustaria tener más respeto por mí mismo/a | 1 | 2 | 3 | 4 | 5 | 0 |
| i) A veces creo que no soy bueno/a en absoluto | 1 | 2 | 3 | 4 | 5 | 0 |
| j) De vez en cuando me siento realmente inútil | 1 | 2 | 3 | 4 | 5 | 0 |

**A partir del sumatorio de los 10 items (transformados en la misma escala) se genera la variable continua SUMAautoestima. A mayor puntuación, mayor autoestima.**

**Los items en rojo son inversos, por lo que se han tratado con la escala invertida.**

1. **¿Cómo se ajustan a ti las siguientes afirmaciones?** (Elige una opción en cada categoría) **RELIGION**

|  | ***Se aplica muy bien a mí*** | ***Se aplica bastante bien a mí*** | ***Se aplica mal a mí*** | ***Se aplica muy mal a mí*** | No contesta |
| --- | --- | --- | --- | --- | --- |
| Creo en Dios | 4 | 3 | 2 | 1 | 0 |
| Mi fe es importante para mí | 4 | 3 | 2 | 1 | 0 |
| Ruego a Dios con regularidad | 4 | 3 | 2 | 1 | 0 |
| Yo leo regularmente los textos sagrados de mi fe | 4 | 3 | 2 | 1 | 0 |
| Regularmente asisto a los servicios religiosos | 4 | 3 | 2 | 1 | 0 |
| Participo regularmente en actividades religiosas diferentes de los servicios | 4 | 3 | 2 | 1 | 0 |
| Podría obtenir apoyo de Dios si lo necesitara | 4 | 3 | 2 | 1 | 0 |
| He buscado el apoyo de Dios cuando lo he necesitado | 4 | 3 | 2 | 1 | 0 |
| Mis mejores amigos/as son religiosos/as | 4 | 3 | 2 | 1 | 0 |
| La mayoría de mis conocidos son religiosos/as | 4 | 3 | 2 | 1 | 0 |
| Mi madre (adoptiva/madrastra) es religiosa | 4 | 3 | 2 | 1 | 0 |
| Mi padre (adoptante/padrastro) es religioso | 4 | 3 | 2 | 1 | 0 |

A partir de estos items se calcula **SUMAreligion**, que es la suma de las puntuaciones de los items anteriores. A mayor puntuación, mayor religiosidad.

1. **¿Consumes diariamente las siguientes bebidas?** (Elige una opción en cada categoría)

|  | ***Sí*** | No | No contesta |
| --- | --- | --- | --- |
| Café CAFE | 1 | 2 | 9 |
| Té TE | 1 | 2 | 9 |
| Bebidas cola (como Coke, Pepsi, etc) COLAS | 1 | 2 | 9 |
| Bebidas energéticas que continenen cafeína (Red bull, Nocco, Monster) ENERGETICAS | 1 | 2 | 9 |

1. **¿Alguna de las siguientes personas fuma tabaco diariamente?** (Elige una opción en cada categoría)

|  | ***Sí*** | No | No contesta |
| --- | --- | --- | --- |
| Padre PADREFUMA | 1 | 0 | 9 |
| Madre MADREFUMA | 1 | 0 | 9 |
| Hermano/a (uno o más) HERMANOFUMA | 1 | 0 | 9 |
| Mejor amigo/a AMIGOFUMA | 1 | 0 | 9 |

1. **¿Has fumado alguna vez en tu vida?** (Elige solo una opción)

|  | ***Sí*** | No | No contesta |
| --- | --- | --- | --- |
| Cigarrillos CIGARRILLOS | 1 | 0 | 9 |
| CIGARRILLOS ELECTRÓNICOS VAPEO | 1 | 0 | 9 |
| Cachimba/pipa de agua CACHIMBA | 1 | 0 | 9 |

1. **¿Qué cantidad de cigarrillos, cigarrillos electrónicos/vapeo y/o cachimba/pipa de fumar has fumado, de media, durante los últimos 30 días?** (Elige solo una opción)

|  | ***Ninguna*** | ***Menos de un cigarrillo por semana*** | ***Menos de un cigarrillo por día*** | ***1-5 cigarrillos por día*** | 6-10 cigarrillos por día | Más de 10 cigarrillos por día | No ha fumado nunca | No contesta |
| --- | --- | --- | --- | --- | --- | --- | --- | --- |
| Cigarrillos CIGARRILLOS30 | 0 | 1 | 2 | 3 | 4 | 5 | 6 | 9 |
| Cigarrillos electrónicos/vapeo VAPEO30 | 0 | 1 | 2 | 3 | 4 | 5 | 6 | 9 |
| Cachimba/pipa de fumar CACHIMBA30 | 0 | 1 | 2 | 3 | 4 | 5 | 6 | 9 |

**CIGARRILLOS30_D 🡪 Se construye una nueva variable dicotòmica. 0=No; 1=Sí; 9=NC.**

**VAPEO30_D 🡪 Se construye una nueva variable dicotòmica. 0=No; 1=Sí; 9=NC.**

**CACHIMBA30_D 🡪 Se construye una nueva variable dicotòmica. 0=No; 1=Sí; 9=NC.**

1. **¿Alguna de las siguientes personas se emborracha al menos una vez a la semana? OPCIONAL** (Elige una opción en cada categoría)

|  | ***Sí*** | No | No contesta |
| --- | --- | --- | --- |
| Padre PADREBORRACHERA | 1 | 2 | 9 |
| Madre MADREBORRACHERA | 1 | 2 | 9 |
| Hermano/a (uno o más) HERMANOBORRACHERA | 1 | 2 | 9 |
| Mejor amigo/a AMIGOBORRACHERA | 1 | 2 | 9 |

1. **Has consumido alguna bebida de alcohol de cualquier tipo en tu vida? ALCOHOLVIDA**

Sí 1

No 2

NC 9

1. **¿Si has consumido una bebida de alcohol de cualquier tipo durante los últimos 30 días, con qué frecuencia lo has hecho? ALCOHOL30**

Nunca 0

1-2 veces 1

3-9 veces 2

10-20 veces 3

Más de 20 veces 4

NC 9

**ALCOHOL30_D 🡪 Se construye una nueva variable dicotòmica. 0=No; 1=Sí; 9=NC.**

1. **¿Con qué frecuencia, si es que lo has hecho, has tomado 4 (cuatro) o más bebidas alcohólicas (por ejemplo, cerveza, vino, licores, chupitos) dentro del plazo de dos horas o más, en los últimos 30 días?** (Elige solo una opción) **BINGEDRINKING30**

Nunca 0

1-2 veces 1

3-9 veces 2

10-20 veces 3

Más de 20 veces 4

NC 9

**BINGEDRINKING30_D 🡪 Se construye una nueva variable dicotòmica. 0=No; 1=Sí; 9=NC.**

1. **¿Te has emborrachado alguna vez en la vida? BORRACHERAVIDA**

Sí 1

No 2

NC 9

1. **¿Si te has emborrachado en los últimos 30 días, con qué frecuencia lo has hecho? BORRACHERA30**

Nunca 0

1-2 veces 1

3-9 veces 2

10-20 veces 3

Más de 20 veces 4

NC 9

**BORRACHERA30_D 🡪 Se construye una nueva variable dicotòmica. 0=No; 1=Sí; 9=NC.**

1. **¿Bebes alcohol en los sitios siguientes?** (Elige una opción en cada categoría)

|  | ***Nunca*** | ***Rara vez*** | ***A veces*** | ***A menudo*** | No contesta |
| --- | --- | --- | --- | --- | --- |
| En tu casa CASAALCOHOL | 1 | 2 | 3 | 4 | 9 |
| En la casa de otros CASAOTROSALCOHOL | 1 | 2 | 3 | 4 | 9 |
| En el centro comercial CENTROCOMERCIALALCOHOL | 1 | 2 | 3 | 4 | 9 |
| Al aire libre, por ejemplo: en la calle, en el parque, etc. CALLEALCOHOL | 1 | 2 | 3 | 4 | 9 |
| En un club/bar/pub BARALCOHOL | 1 | 2 | 3 | 4 | 9 |
| En un club juvenil CLUBALCOHOL | 1 | 2 | 3 | 4 | 9 |
| Después de las pruebas de entrenamiento DESPUESENTRENOALCOHOL | 1 | 2 | 3 | 4 | 9 |

1. **¿Has consumido cannabis alguna vez en tu vida? CANNABISVIDA**

Sí 1

No 2

NC 9

1. **¿Si has consumido cannabis durante los últimos 30 días, con qué frecuencia lo has hecho? CANNABIS30**

Nunca 0

1-2 veces 1

3-9 veces 2

10-20 veces 3

Más de 20 veces 4

NC 9

**CANNABIS30_D 🡪 Se construye una nueva variable dicotòmica. 0=No; 1=Sí; 9=NC.**

1. **Alguna de les siguientes personas consume cannabis?** (Elige una opción en cada categoría)

|  | ***Sí*** | No | No contesta |
| --- | --- | --- | --- |
| Padre PADRECANNABIS | 1 | 0 | 9 |
| Madre MADRECANNABIS | 1 | 0 | 9 |
| Hermano/a (uno o más) HERMANOCANNABIS | 1 | 0 | 9 |
| Mejor amigo/a AMIGOCANNABIS | 1 | 0 | 9 |

1. **Has consumido alguna vez las siguientes sustancias?** (Elige una opción en cada categoría)

|  | ***Sí*** | No | No contesta |
| --- | --- | --- | --- |
| Pastillas para dormir o tranquilizantes TRANQUILIZANTES | 1 | 0 | 9 |
| Cannabis (hachís o marihuana) CANNABIS | 1 | 0 | 9 |
| Anfetaminas (speed) ANFETAMINAS | 1 | 0 | 9 |
| LSD (ácido) LSD | 1 | 0 | 9 |
| Éxtasis (E-tablets) EXTASIS | 1 | 0 | 9 |
| Cocaína COCAINA | 1 | 0 | 9 |
| Heroína HEROINA | 1 | 0 | 9 |
| Relevina RELEVINA | 1 | 0 | 9 |
| Setas SETAS | 1 | 0 | 9 |
| Inhalantes (p. ej. Cola) INHALANTES | 1 | 0 | 9 |
| Esteroides anabolitzantes ESTEROIDES | 1 | 0 | 9 |
| Drogas orgánicas/hierbas DROGASORGANICAS | 1 | 0 | 9 |
| Medicamentos opiacios sin receta médica (por ejemplo, Codeína, Morfina, Metadona, Fentanil, Oxicontina, Hidrocodona y otros) OPIACEOS | 1 | 0 | 9 |
| Medicamento para el TDAH sin recepta médica (por ejemplo; Ritalin, Concerta, Rubifen) MEDICAMENTOTDAH | 1 | 0 | 9 |
| Nuevas sustancias (keta, spice, miau miau, flakka, superman...) NUEVASSUSTANCIAS | 1 | 0 | 9 |

**Se recodifican todas las anteriores sustancias (excepto Relevina) con 1=si y 0=no. Se suman para obtenir la nueva variable POLICONSUMO (no incluye ni alcohol ni tabaco).**

1. **¿A qué edad (si es que lo has hecho) has consumido o hecho alguna de las siguientes situaciones por primera vez?** (Elige una opción por cada categoría)

|  | ***Nunca*** | ***11 años o menos*** | ***12 años*** | ***13 años*** | 14 años | 15 o más | No contesta |
| --- | --- | --- | --- | --- | --- | --- | --- |
| Consumir alcohol EDADALCOHOL | 0 | 1 | 2 | 3 | 4 | 5 | 9 |
| Me emborraché EDADBORRACHERA | 0 | 1 | 2 | 3 | 4 | 5 | 9 |
| Fumar un cigarrillo EDADCIGARRILLO | 0 | 1 | 2 | 3 | 4 | 5 | 9 |
| Fumar cigarrillos diariamente EDADTABACODIARIO | 0 | 1 | 2 | 3 | 4 | 5 | 9 |
| Fumar cannabis (hachís / marihuana) EDADCANNABIS | 0 | 1 | 2 | 3 | 4 | 5 | 9 |
| Fumar cigarrillos Electrónicos EDADVAPEO | 0 | 1 | 2 | 3 | 4 | 5 | 9 |

1. **¿Cuántos de tus amigos crees que hace lo siguiente?** (Elige una opción por cada categoría)

|  | ***Ninguno*** | Algunos | La mayoría | No contesta |
| --- | --- | --- | --- | --- |
| Fumar cigarrillos AMIGOSTABACO | 1 | 2 | 3 | 9 |
| Beber alcohol (cerveza, vino o licores) AMIGOSALCOHOL | 1 | 2 | 3 | 9 |
| Emborracharse al menos una vez al mes AMIGOSBORRACHERA | 1 | 2 | 3 | 9 |
| Fumar hachís o marihuana AMIGOSCANNABIS | 1 | 2 | 3 | 9 |

1. **Haces ejercicio o practicas deporte, fuera del instituto y fuera de un club/equipo? PRACTICADEPORTE**

Nunca 0

Sí, de vez en cuando 1

Sí, a menudo 2

Sí, cada día 3

NC 9

1. **Practicas deporte (practicar o competir) con un club/equipo deportivo? DEPORTECLUB**

Sí 1

No 2

NC 9

1. **¿Haces actividades extraescolares supervisadas por adultos?** (Elige una opción por cada categoría)

|  | ***Sí*** | No | No contesta |
| --- | --- | --- | --- |
| Grupo de aprendizaje de idiomes IDIOMAS | 1 | 0 | 9 |
| Música, arte, teatro o danza ARTES | 1 | 0 | 9 |
| Otras actividades extracurriculares (por ejemplo; ajedrez, etc.) OTRASEXTRA | 1 | 0 | 9 |
| Actividades Deportivas DEPORTES | 1 | 0 | 9 |
| Profesor/a particular PROFPARTICULAR | 1 | 0 | 9 |

1. **¿Actualmente perteneces a alguna entidad o asociación de manera voluntaria?** (Elige una opción)

|  | ***Sí*** | No | No contesta |
| --- | --- | --- | --- |
| Esplai ESPLAI | 1 | 0 | 9 |
| Entidad Deportiva CLUBESPORTIU | 1 | 0 | 9 |
| Entidad cultural ENTITATCULTURAL | 1 | 0 | 9 |
| Entidad de voluntariado VOLUNTARIAT | 1 | 0 | 9 |

**ASOCIACIONISMO 🡪 Se construye una nueva variable dicotòmica 1=Si y 0=No. El valor 1 se da si ha puntuado con este mismo valor a alguna de las variables 52.1 a 52.4**

**Las siguientes preguntas son sobre uso de Internet.**

1. **¿Consideras que estás bien/mal informado respecto a las consecuencias del consumo de sustancias? INFORMADO**

Muy bien informado 5

Bien informado 4

Tengo dudas 3

Mal informado 2

Muy mal informado 1

NC 9

1. **¿A través de qué medios te informas prioritariamente sobre el consumo de sustancias?**

|  | ***Sí*** | No | No contesta |
| --- | --- | --- | --- |
| A través de Internet INFOINTERNET | 1 | 0 | 9 |
| A través de charlas en el centro educativo INFOCENTRO | 1 | 0 | 9 |
| A través de mis pedres INFOPADRES | 1 | 0 | 9 |
| A través de mis hermanos/as INFOHERMANOS | 1 | 0 | 9 |
| A través de mis amigos/as INFOAMIGOS | 1 | 0 | 9 |
| A través de medios de comunicación INFOMEDIOS | 1 | 0 | 9 |

1. **¿A través de qué medios te gustaria recibir información sobre drogas?**

|  | ***Sí*** | No | No contesta |
| --- | --- | --- | --- |
| Personal sociosanitario SOCIOSANITARIOINFO | 1 | 0 | 9 |
| Cuerpos de Seguridad FFSSINFO | 1 | 0 | 9 |
| Llibros y/o trípticos informativos DOCUMENTOSINFO | 1 | 0 | 9 |
| Padre/madre/familiares FAMILIAINFO | 1 | 0 | 9 |
| Personas que han tenido contacto con elles CONSUMIDORESINFO | 1 | 0 | 9 |
| Charlas o cursos sobre el tema CURSOSINFO | 1 | 0 | 9 |
| Profesores/as PROFESINFO | 1 | 0 | 9 |
| A través de Internet INTERNETINFO | 1 | 0 | 9 |
| Medios de comunicación MEDIOSINFO | 1 | 0 | 9 |
| Amigo/a o conocido/a o compañero/a de estudiós PARESINFO | 1 | 0 | 9 |

1. **¿Has recibido alguna charla o sesión sobre drogas en el centro donde estudias? CENTROINFORMA**

Sí 1

No 2

NS 8

NC 9

1. **Si la respuesta es afirmativa, valora la utilidad de la misma: UTILIDADINFO**

Muy útil 1

Bastante útil 2

No me ha servido de mucho 3

No me ha servido de nada 4

NC 9

1. **Ahora nos gustaria saber tu opinión sobre los problemas de salud o de otro tipo que puede significar realizar cada una de las siguientes conductas.**

|  | ***Pocos problemas o nunguno*** | Bastantes o muchos problemas | No sabes |
| --- | --- | --- | --- |
| Fumar un paquete diario PAQUETEDIARIO | 1 | 2 | 9 |
| Fumar de 1 a 5 cigarrillos diarios TABACODIARIO | 1 | 2 | 9 |
| Tomarse 5 o 6 cañas/copas de bebidas alcohólicas el fin de semana ALCOHOLSEMANA | 1 | 2 | 9 |
| Tomar 1 o 2 cañas/copas de bebidas alcohólicas cada día ALCOHOLDIARIO | 1 | 2 | 9 |
| Consumir tranquilizantes/sedantes o somníferos habitualment TRANQILIZANTEHABITUAL | 1 | 2 | 9 |
| Fumar hachís/marihuana (cannabis) habitualment CANNABISHABITUAL | 1 | 2 | 9 |
| Consumir cocaína en polvo habitualmente COCAINAHABITUAL | 1 | 2 | 9 |
| Consumir éxtasis habitualment EXTASISHABITUAL | 1 | 2 | 9 |
| Consumir heroína habitualment HEROINAHABITUAL | 1 | 2 | 9 |

1. **También nos gustaría saber tu opinión sobre los problemas (de salud o de otro tipo) que puede significar realizar alguna vez (una vez al mes o menos frecuentemente) cada una de les siguientes conductas.**

|  | ***Pocos problemas o nunguno*** | Bastantes o muchos problemas | No sabes |
| --- | --- | --- | --- |
| Fumar cigarrillos Electrónicos VAPEOALGUNAVEZ | 1 | 2 | 9 |
| Fumar hachís/marihuana (cannabis) alguna vez CANNABISALGUNAVEZ | 1 | 2 | 9 |
| Consumir cocaína en polvo alguna vez COCAINAALGUNAVEZ | 1 | 2 | 9 |
| Fumar base (cocaína base, crack) alguna vez BASEALGUNAVEZ | 1 | 2 | 9 |
| Consumir éxtasis alguna vegada EXTASISALGUNAVEZ | 1 | 2 | 9 |
| Consumir amfetaminas o speed alguna vez ANFETAMINASALGUNAVEZ | 1 | 2 | 9 |
| Consumir alucinógenos (LSD, tripis o setas mágicas) alguna vez ALUCINÓGENOSALGUNAVEZ | 1 | 2 | 9 |
| Consumir heroína alguna vez HEROINAALGUNAVEZ | 1 | 2 | 9 |
| Inyectarse drogas alguna vez INYECTARSEALGUNAVEZ | 1 | 2 | 9 |
| Consumir GHB alguna vez GHBALGUNAVEZ | 1 | 2 | 9 |
| Consumir metamfetamina alguna vez METAANFETAALGUNAVEZ | 1 | 2 | 9 |

1. **¿Con qué frecuencia te ha resultado difícil dejar de usar Internet cuando estabas conectado/a?** (Elige una opción) **USOINTERNET**

|  | ***Nunca*** | Rara vez | Alguna vez | A menudo | Frecuentemente | No contesta |
| --- | --- | --- | --- | --- | --- | --- |
| Has seguido usando Internet a pesar de tu intención de querer parar | 0 | 1 | 2 | 3 | 4 | 9 |
| Tu padre/madre o amigos/as te han dicho que deberías pasar menos tiempo en Internet | 0 | 1 | 2 | 3 | 4 | 9 |
| Has preferido conectarte a Internet en vez de pasar el tiempo con otros (amigos/as o padres) | 0 | 1 | 2 | 3 | 4 | 9 |
| Duermes menos por estar conectado/a a Internet | 0 | 1 | 2 | 3 | 4 | 9 |
| Te encuentras pensando en Internet, aunque no estés conectado | 0 | 1 | 2 | 3 | 4 | 9 |
| Estas deseando conectarte a Internet | 0 | 1 | 2 | 3 | 4 | 9 |
| Piensas que deberías usar menos Internet | 0 | 1 | 2 | 3 | 4 | 9 |
| Has intentado pasar menos tiempo conectado a Internet y no lo has conseguido | 0 | 1 | 2 | 3 | 4 | 9 |
| Intentas terminar tu trabajo a toda prisa para conectarte a Internet | 0 | 1 | 2 | 3 | 4 | 9 |
| Has descuidado tus obligaciones diarias (como el trabajo, la escuela o la vida familiar) porque prefieres conectarte a Internet | 0 | 1 | 2 | 3 | 4 | 9 |
| Te conectas a Internet cuando estás “de bajón” | 0 | 1 | 2 | 3 | 4 | 9 |
| Te conectas a Internet para olvidar tus penas o sentimientos negativos | 0 | 1 | 2 | 3 | 4 | 9 |
| Te sientes inquieto/a, frustrado/a o irritado/a si no puedes usar Internet? | 0 | 1 | 2 | 3 | 4 | 9 |

**CIUS🡪 Se construye una nueva variable dicotòmica 1=Cius y 0=No Cius. El valor 1 se alcanza a partir de la puntuación mayor o igual a 26 en la suma de los 13 items anteriores.**

**Las siguientes preguntas son sobre uso de videojuegos.**

1. **En los útlimos 12 meses, ¿con qué frecuencia has realizado cada una de las siguientes actividades? JUEGOONLINEAÑO**

|  | ***Nunca*** | De 1 a 3 días al año | De 1 a 3 días al mes | De 1 a 4 días por semana | De 5 a 7 días por semana | No contesta |
| --- | --- | --- | --- | --- | --- | --- |
| Jugar a videojuegos | 1 | 2 | 3 | 4 | 5 | 9 |
| Jugar a eSports o deportes electónicos | 1 | 2 | 3 | 4 | 5 | 9 |
| Participar como espectador en eSports o deportes electrónicos | 1 | 2 | 3 | 4 | 5 | 9 |

1. **En los útlimos 12 meses, ¿cuántas hores de media dedicas a estas actividades en un día? HORASDIARIASJUEGOONLINE**

|  | ***No lo he hecho*** | Menos de 2 horas | De 2 a 5 horas | De 6 a 8 horas al día | Más de 8 horas al día | No contesta |
| --- | --- | --- | --- | --- | --- | --- |
| Jugar a videojuegos | 1 | 2 | 3 | 4 | 5 | 9 |
| Jugar a eSports o deportes electrónicos | 1 | 2 | 3 | 4 | 5 | 9 |
| Participar como espectador en eSports o deportes electrónicos | 1 | 2 | 3 | 4 | 5 | 9 |

1. **En los útlimos 12 meses, ¿cuánto dinero te has gastado en total en un videojuego para mejorar tu posición, tu personaje, accesorios, imagen...? DINEROVIDEOJUEGO**

Nada 1

Entre 1 y 50 euros 2

Entre 51 y 100 euros 3

Más de 100 4

NC 9

1. **Lee las afirmaciones siguientes respecto al uso de videojuegos. Indica si te han ocurrido las siguientes situaciones en los últimos 12 meses UCVIDEOJUEGO (Uso Compulsivo Videojuego)**

|  | ***Nunca*** | Rara vez | Alguna vez | A menudo | Frecuentemente | No contesta |
| --- | --- | --- | --- | --- | --- | --- |
| ¿Estás frecuentemente pensando en los videojuegos o en cómo vas a jugar al siguiente juego o la siguiente partida? | 0 | 1 | 2 | 3 | 4 | 9 |
| ¿Te sientes irritado/a, inquieto/a o triste si no puedes jugar a los videojuegos? | 0 | 1 | 2 | 3 | 4 | 9 |
| ¿Necesitas estar cada vez más tiempo jugando a los videojuegos? | 0 | 1 | 2 | 3 | 4 | 9 |
| ¿Has intentado pasar menos tiempo jugando a videojuegos y no lo has conseguido? | 0 | 1 | 2 | 3 | 4 | 9 |
| ¿Por jugar a los videojuegos has perdido interés por otras actividades que hacías antes? | 0 | 1 | 2 | 3 | 4 | 9 |
| ¿Contínuas jugando mucho con los videojuegos a pesar de conocer las consecuencias negativas que eso tiene? | 0 | 1 | 2 | 3 | 4 | 9 |
| ¿Has engañado a tus familiares u otras personas sobre le tiempo que pasas jugando a videojuegos? | 0 | 1 | 2 | 3 | 4 | 9 |
| ¿Has jugado a videojuegos para sentirte mejor cuando estás “de bajón” o cuando estás enfadado/a o nervioso/a? | 0 | 1 | 2 | 3 | 4 | 9 |
| ¿Has puesto en peligro o perdido alguna amistad o relación importante o has dejado de lado los estudios por jugar a videojuegos? | 0 | 1 | 2 | 3 | 4 | 9 |

1. **En los últimos 12 meses, ¿con qué frecuencia has jugado dinero? FRECUENCIADINEROONLINE**

|  | Un día al mes o menos | ***2-4*** días ***al mes*** | ***2-3*** días ***a la semana*** | ***4-5*** días ***a la semana*** | ***6 o más*** días ***a la semana*** | No he jugado dinero en los últimos 12 meses | Nunca he jugado dinero |
| --- | --- | --- | --- | --- | --- | --- | --- |
| En Internet (online) | 1 | 2 | 3 | 4 | 5 | 6 | 7 |
| Fuera d’e nternet (presencial) | 1 | 2 | 3 | 4 | 5 | 6 | 7 |

1. **¿Qué edad tenías la primera vez que jugaste dinero?**

|  | ***Nunca*** | ***11 años o menos*** | ***12 años*** | ***13 años*** | 14 años | 15 o más | No contesta |
| --- | --- | --- | --- | --- | --- | --- | --- |
| En Internet (online) | 0 | 1 | 2 | 3 | 4 | 5 | 9 |
| Fuera de Internet (presencial) | 0 | 1 | 2 | 3 | 4 | 5 | 9 |
